# Supplementary material for: Anti-Influenza A Potential of Tagetes erecta Linn. Extract Based on Bioinformatics Analysis and In Vitro Assays
Source: Int J Mol Sci. 2024 Jun 27;25(13):7065. doi: 10.3390/ijms25137065 (PMC11241564; doi:10.3390/ijms25137065)
Supplement: Supplementary file 1 [file ijms-25-07065-s001.zip › ijms-3018460-supplementary/Supp.Figures.ijms.pdf]

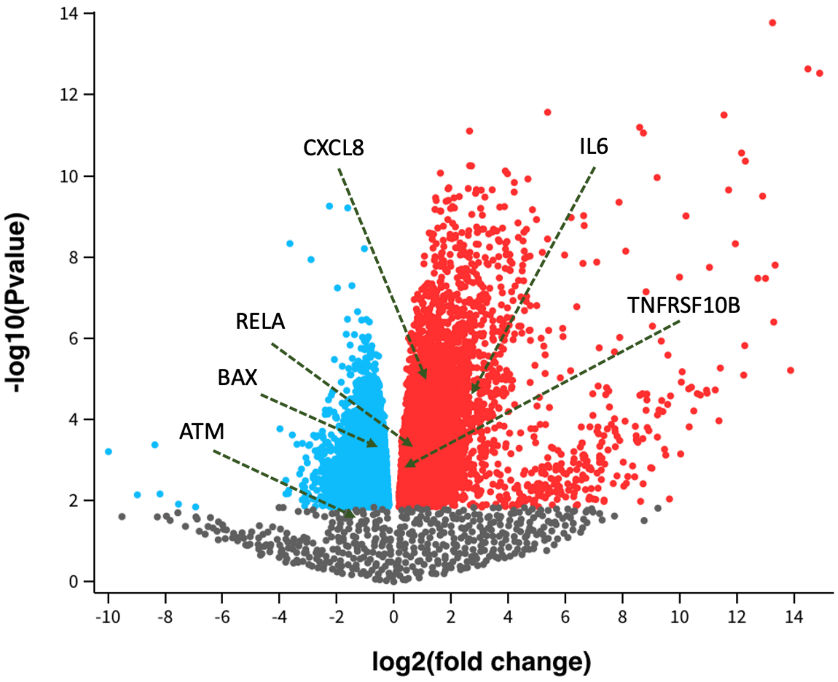

Supplementary Figure S1. Volcano plot of differentially expressed genes between H1N1 and healthy control samples. The locations of six target genes are shown by arrows. The TGFB1 gene was not included in the differentially expressed gene list.

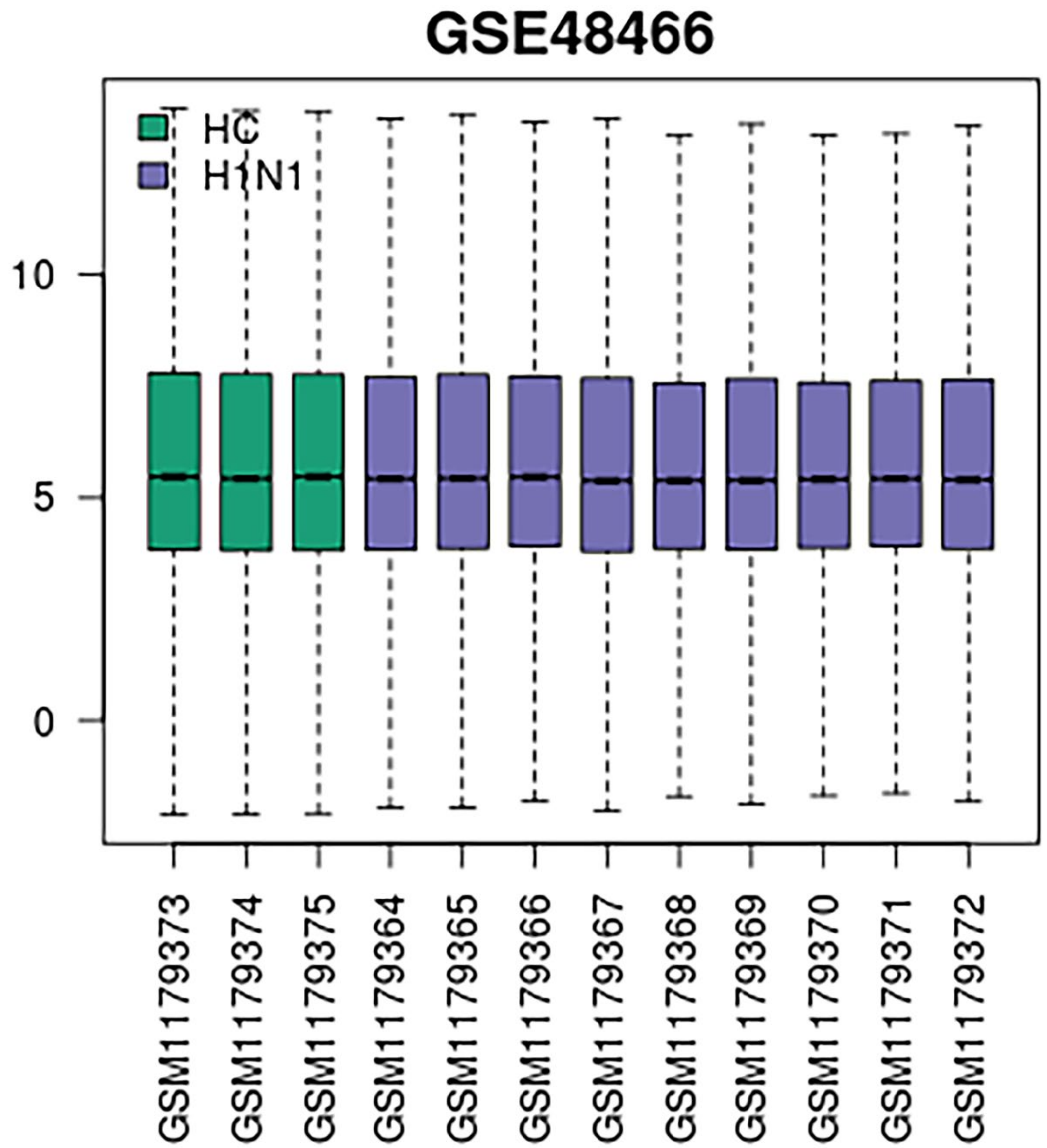

Supplementary Figure S2. Box plot of differentially expressed genes between H1N1 and healthy control samples.

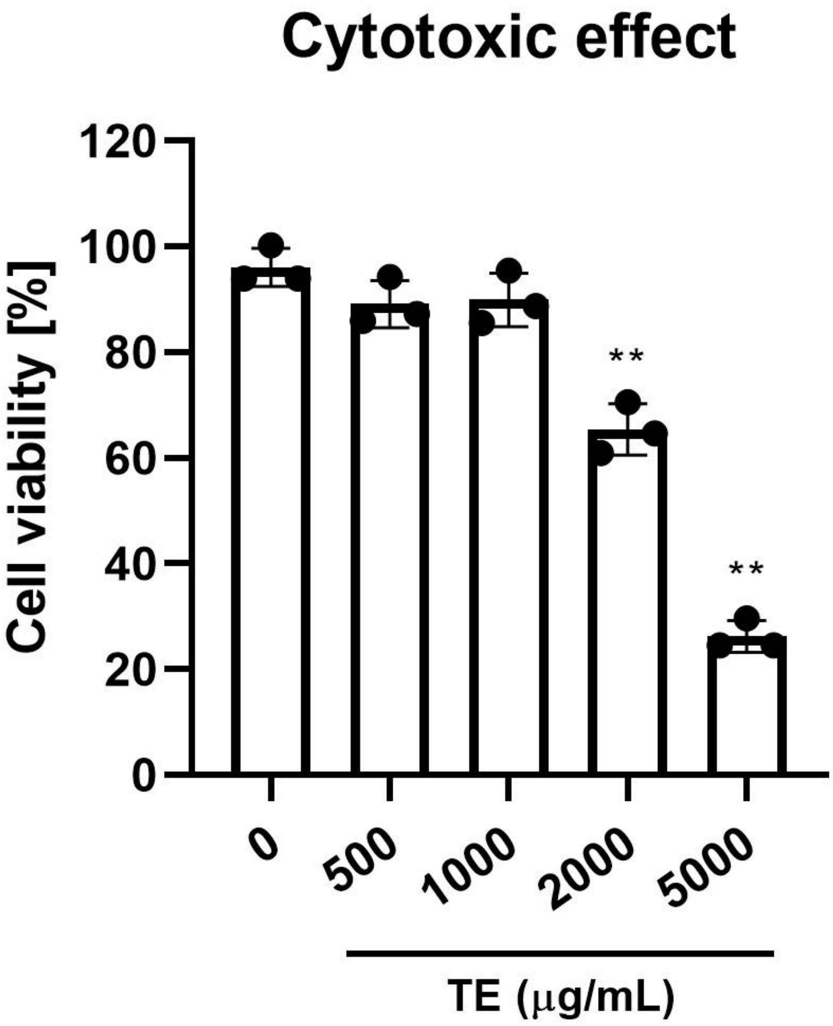

Supplementary Figure S3: Cytotoxic effect of TE extract in MDCK cells. ***P < 0.01* (compared with the untreated control group, 0 μg/mL).
