# Supplementary material for: Anti-Influenza A Potential of Tagetes erecta Linn. Extract Based on Bioinformatics Analysis and In Vitro Assays
Source: Int J Mol Sci. 2024 Jun 27;25(13):7065. doi: 10.3390/ijms25137065 (PMC11241564; doi:10.3390/ijms25137065)
Supplement: Supplementary file 1 [file ijms-25-07065-s001.zip › ijms-3018460-supplementary/Supplementary Table 1.pdf]

Supplementary Table *Tagetes erecta* Linn flower part plant extract composition

**Chemical**	**Plant Part**
2,4-METHYLENE-CHOLESTEROL	Flower
2-CRYPTOXANTHIN	Flower
28-ISOFUCOSTEROL	Flower
4-BETA-METHYLERGOSTA-7,24(28)-DIEN-3-BETA-OL	Flower
4-BETA-METHYLSTIGMASTA-7,24(28)-DIEN-3BETA-OL	Flower
6-HYDROXYKAEMPFEROL-7-O-GLUCOSIDE	Flower
8-HYDROXYQUERCETAGETIN	Flower
ALPHA-CAROTENE	Flower
ANTHERAXANTHIN	Flower
BETA-CAROTENE	Flower
BETA-SITOSTEROL	Flower
CAMPESTEROL	Flower
CHOLESTEROL	Flower
DITHIENYLACETYLENE	Flower
HELENIEN	Flower
KAEMPFERITRIN	Flower
LUTEIN	Flower
LUTEIN-DIMYRISTATE	Flower
LUTEIN-DIPALMITATE	Flower
LUTEIN-MONOMYRISTATE	Flower
PHYTOFLUENE	Flower
PYRETHRINS	Flower
QUERCETAGETIN	Flower
QUERCETAGETIN-3-GLUCOSIDE	Flower
QUERCETAGETIN-7-GLUCOSIDE	Flower
QUERCETAGITRIN	Flower
STIGMASTEROL	Flower
TAGETIIN	Flower
